# Supplementary material for: Are we walking the talk of participatory Indigenous health research? A scoping review of the literature in Atlantic Canada
Source: PLoS One. 2021 Jul 27;16(7):e0255265. doi: 10.1371/journal.pone.0255265 (PMC8315539; doi:10.1371/journal.pone.0255265)
Supplement: S1 Table — (DOCX) [file pone.0255265.s001.docx]

**Search Terms used in the Electronic Databases to Identify Articles for the Scoping Review of Indigenous Health Research Conducted in Atlantic Canada from 2001-June 2020.**

| **Keywords and Subject Headings for Each Search Concept** | | | |
| --- | --- | --- | --- |
| **Indigenous Peoples in Atlantic Canada** (each of these terms was combined with the Boolean OR) | **AND** | **Geographic regions and Indigenous communities in Atlantic Canada** (each of the below terms were combined with the Boolean OR) | **Health Research** (each of the below terms were combined with the Boolean OR)  *Note: these terms were only used for databases not related to health, including: CAB Abstracts, Bibliography of Native North Americans, and Academic Search Premier |
| Aboriginal, First Nation, Native Canadian, Indigenous, Inuit, Innu, Metis, Métis, Inuk, Micmac, Mi’kmaq , Mi’kmak, Mi’kmaw, Migmaw, Mi’gmaq, Micmaw, Peskotomuhtaki, Passamaquoddy, L’nu, Beothuk, Maliseet, Wolastoqiyik, Malecite, Amerindian, Autochtone, Urban Indian, Montagnais, Naskapi, Eskimo, Algonquin, Alonguian, Abenaki, on reserve, off reserve |  | Broad regional/provincial names: Eastern Canada, Maritimes, Atlantic Canada, Nova Scotia, Atlantic provinces, New Brunswick, Newfoundland, Labrador, PEI, Prince Edward Island, Mi’kma’ki, Wabanaki, Eastern Woodland, Woodlands  Nova Scotia: Cape Breton, Nouvelle Ecosse, Unama’ki, Pictou Landing First Nation, Puksaqte’kne’katik*,* Bear River First Nation, L’setkuk, Boat Harbour, Annapolis Valley First Nation, Kampalijek, Cambridge Reserve, Chapel Island, Eskasoni First Nation, Eskisoqnik*,* Sipekne’katik First Nation*,* Paq’tnkek First Nation, Paqtnkek*,* Potlotek First Nation, Glooscap First Nation, Pesikitk*,* Acadia First Nation, Malikiaq, Gold River Reserve, Horton Reserve, Shubenacadie First Nation, Indian Brook Reserve, Wagmatcook First Nation, Wagmitkuk*,* Waycobah First Nation, We’koqma’q*,* Millbrook First Nation, We’kopekwitk*,* Membertou First Nation, Maupeltuk*,* New Ross Reserve, Pennal Reserve, St Croix Reserve, Summerside Reserve, Sydney Reserve, Truro Reserve, We’koqma’q, Whycocomagh, Wildcat Reserve, Yarmouth Reserve, Malagawatch, Medway River, Merigomish Harbour Reserve, Musquodoboit, Beaver Lake, Caribou Marsh, Franklin Manor, Margaree, Ponhook Lake, Wallace Hills, Welnek, Cole Harbour, Sheet Harbour, Beaver Dam, Pomquet, Afton, Franklin Manor, Summerside, Fisher’s Grant, K’jiptuk, Unamaki  Newfoundland and Labrador: Newfoundland, Labrador, NFLD, Nain, Rigolet, Hopedale, Utshimasset, Davis Inlet, Miawpukek First Nation, Mi’kamawey Mawi’omi or Makkovik, Nunatsiavut, Urahimassit, NunatuKavut, Cartwright, Black Tickle, Norman’s Bay, Port Hope Simpson, Charlottetown, St. Lewis, Fox Harbour, Mary’s Harbour, Pinsent’s Arm, Lodge Bay, William’s Harbour, Natuashish, Mushuau Innu First Nation, Sheshatshiu Innu First Nation, Sheshatshiu Innu First Nation  New Brunswick: Nouveau Brunswick, Big Hole Tract, Metepenagiag, Eel Ground First Nation, Natuaqanek, Burnt Church, Devon Reserve, St Mary?s First Nation, Eel River Bar First Nation, Oqpi’kanjik, Fort Folly, Amlamkuk Kwesawe’k, Indian Point Reserve, Indian Island First Nation, L’nui Menikuk, Indian Ranch Reserve, Kingsclear First Nation, Pabineau First Nation, Ke’kwapskuk, Red Bank Reserve, Soegao Reserve, Tobique First Nation, Woodstock First Nation, Buctouche Micmac, Puktusk, Buctouche Reserve, Buctouche MicMac, Elsipogtog First Nation, Esgenoopetitj First Nation, Eskinuopitijk, Madawaska Maliseet First Nation, Metepenagiag Mi’kmaq Nation, Metepna’kiaq, Oromocto First Nation, Moose Meadows, PokeMouche Reserve, Renous Reserve, Richibucto Reserve, St. Basile, Devon Indian Reserve, Tabusintac Reserve, Pekotomuhkati Nation  PEI: Abegweit First Nation, Epekwitk, Lennox Island First Nation, L’nui Mnikuk, Morell Rear Reserve, Rocky Point Reserve, ScotchFord OR ScotchFort Reserve  Friendship Centers: Mi’kmaq Native Friendship Centre, Mi’kmaw Native Friendship Centre, St. John’s Native Friendship Centre, Under One Sky Friendship Centre, Fredericton Native Friendship Centre | Health keywords: health, medical, dental, medicine, wellness, psychological, psychiatric, wellbeing, “well being”, illness*, disease*, sick*, biomedical  Research subject headings: “research”, “agricultural, “animal experimentation”, “animal models in research”, “archaeological research”, “archival research”, “area studies”, “attention research”, “attrition in research studies”, “biological research”, “business research”, “chemical research”, “citizen science”, “communications research”, “comparative method”, “comparative studies”, “computer assisted research”, “cooperative research”, “demographic research”, “discoveries in science”, “education research”, “empirical research”, “evaluation”, “exceptional children – research”, “experiential research”, “experimental design”, “experiments”, “family research”, “feasibility studies”, “field work (research)”, “flavor research”, “food research”, “government research”, “group work in research”, “group-randomized trials”, “highway research”, “historical research”, “hospitality industry research”, “human experimentation”, “industrial management research”, “industrial organization research”, “industrial research”, “information theory in research”, “intelligence service”, “interdisciplinary research”, “internet research”, “land research”, “language research”, “learning disabilities research”, “legal research”, “library research”, “library science research”, “literary research”, “longitudinal method”, “low temperature research”, “management science research”, “marketing research”, “medical research”, “military research”, “mixed methods research”, “motivation research (marketing)”, “musicology”, “narrative inquiry (research method)”, “naval research”, “observatories”, “operations research”, “packaging research”, “pedagogical content knowledge research”, “physical sciences research”, “physics research”, “pilot projects”, “population research”, “postdoctoral researchers”, “production management (manufacturing) – research”, “proposal writing in research”, “public health research”, “public institutions – research”, “qualitative research”, “quantitative research”, “radioisotopes in research”, “recreation – research”, “reproducible research”, “research & development”, “research bias”, “research implementation”, “research in information science”, “research subjects (persons)”, “retrospective studies”, “scientific development”, “scientific experimentation”, “scientific surveys”, “sexism in research”, “social science research”, “speech research”, “statistical research”, “student research”, “tax research”, “textile research”, “theater research”, “volcanological research”, “wildlife research”, “working papers” |
