# Supplementary figures and images for: Are we walking the talk of participatory Indigenous health research? A scoping review of the literature in Atlantic Canada
Source: PLoS One. 2021 Jul 27;16(7):e0255265. doi: 10.1371/journal.pone.0255265 (PMC8315539; doi:10.1371/journal.pone.0255265)

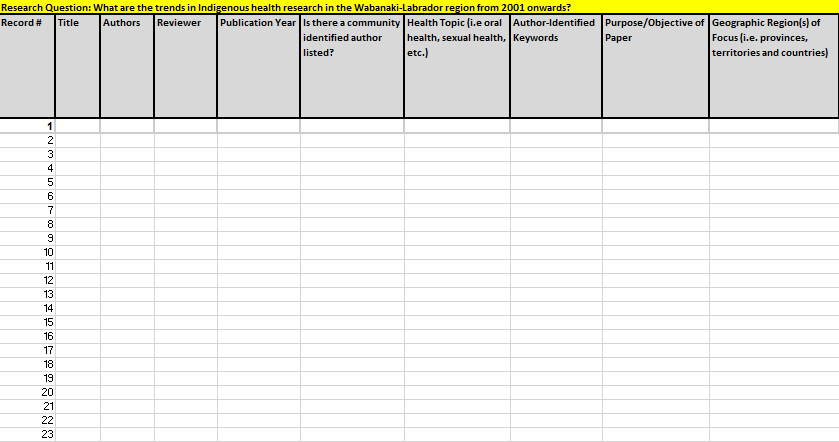

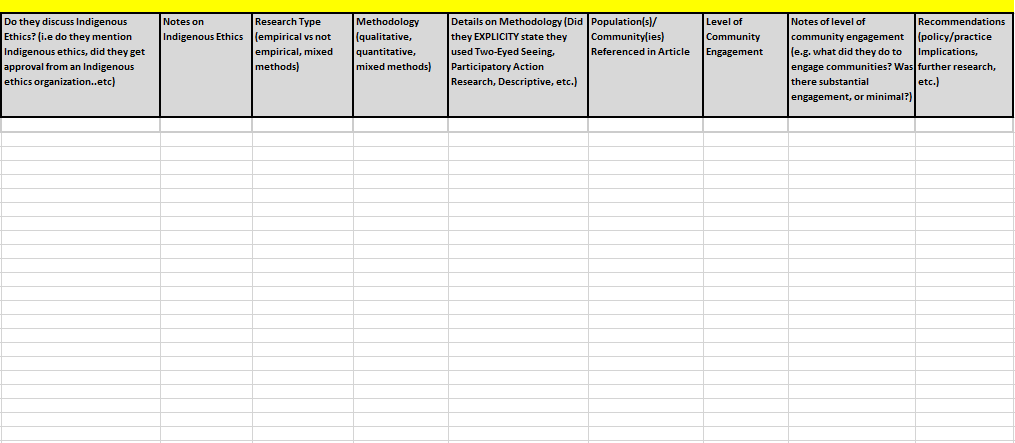
 **Data Charting Form Template**

Supplement: S2 Table — (DOCX) [file pone.0255265.s002.docx]
