# Supplementary material for: Are we walking the talk of participatory Indigenous health research? A scoping review of the literature in Atlantic Canada
Source: PLoS One. 2021 Jul 27;16(7):e0255265. doi: 10.1371/journal.pone.0255265 (PMC8315539; doi:10.1371/journal.pone.0255265)
Supplement: S1 File — (DOCX) [file pone.0255265.s003.docx]

# **Reference List for all Records Included in Analysis**

1. MacDonald NE. Aboriginal children suffer while governments ignore Jordan’s Principle. Cmaj. 2012;184(8):853. Available from: <https://doi.org/10.1503/cmaj.120193>

2. Goldhar C, Bell T, Wolf J. Vulnerability to freshwater changes in the Inuit settlement region of Nunatsiavut, Labrador: A case study from Rigolet. Arctic. 2014;67(1):71–83. Available from: <https://doi.org/10.14430/arctic4365>

3. Spillane NS, Smith GT, Kahler CW. Perceived access to reinforcers as a function of alcohol consumption among one First Nation group. Alcohol Clin Exp Res. 2013;37(SUPPL.1):314–21. Available from: <https://doi.org/10.1111/j.1530-0277.2012.01864.x>

4. Ashawasegai J. Mom and health director fight for Jeremy’s care. Windspeaker [Internet]. 2011;29(5):8. Available from: <http://search.ebscohost.com/login.aspx?direct=true&db=aph&AN=63154801&site=ehost-live>

5. Dobbelsteyn JL. Nursing in First Nations and Inuit communities in Atlantic Canada. Can Nurse. 2006;102(4):32–5.

6. Wright CJ, Sargeant JM, Edge VL, Ford JD, Farahbakhsh K, Shiwak I, et al. How are perceptions associated with water consumption in Canadian Inuit? A cross-sectional survey in Rigolet, Labrador. Sci Total Environ. 2018;618:369–78. Available from: <https://doi.org/10.1016/j.scitotenv.2017.10.255>

7. Andersson N. Affirmative challenges in Indigenous resilience research. Pimatisiwin. 2008;6(2):3–6. Available from: <https://doi.org/10.1002/jts>

8. Zahradnik M, Stewart SH, Sherry SB, Stevens D, Wekerle C. Posttraumatic stress hyperarousal symptoms mediate the relationship between childhood exposure to violence and subsequent alcohol misuse in Mi’kmaq youth. J Trauma Stress. 2011;29(August):293–300. Available from: <https://doi.org/10.1002/jts>

9. Jones J, Cunsolo A, Harper SL. Who is research serving? A systematic realist review of circumpolar environment-related Indigenous health literature. PLoS One. 2018;13(5). Available from: <http://dx.doi.org/10.1371/journal.pone.0196090>

10. Hanrahan M. “Through their own efforts”: Nutrition studies and interventions in early 20-century Northern Newfoundland and Southern Labrador. Cuizine J Can Food Cult. 2016;7(1). Available from: <https://doi.org/10.7202/1037390ar>

11. Watson R, Castleden H, Masuda J, King M, Stewart M. Identifying gaps in asthma education, health promotion, and social support for Mi’kmaq families in Unama’ki (Cape Breton), Nova Scotia, Canada. Prev Chronic Dis. 2012;9(1):1–8. Available from: <https://doi.org/10.5888/pcd9.120039>

12. Sylliboy JR, Hovey RB. Humanizing Indigenous Peoples’ engagement in health care. Cmaj. 2020;192(3):E70–2. Available from: <https://doi.org/10.1503/cmaj.190754>

13. Levi EAM. Maintaining food security in Elsipogtog First Nation [Masters thesis]. Lakehead University; 2007

14. Wright C, Sargeant JM, Edge VL, Ford JD, Farahbakhsh K, Shiwak I, Flowers C, Harper SL. Water quality and health in northern Canada: stored drinking water and acute gastrointestinal illness in Labrador Inuit. 2017. Int. J. Circumpolar Health, *76*(1). Available from: <https://doi.org/10.1080/22423982.2017.1335149>

15. Hanrahan M. Tracing social change among the Labrador Inuit and Inuit-Métis: What does the nutrition literature tell us? Food, Cult Soc. 2008;11(3):315–33. Available from: <https://doi.org/10.2752/175174408X347883>

16. Critchley K, Timmons V, Walton F, Bryanton J, McCarthy M, Taylor J. Building healthy Mi’kmaq communities in Prince Edward Island. Can J Native Stud. 2007;27(1):1–17. Available from: <http://www3.brandonu.ca/cjns/27.1/01Critchly.pdf>

17. Natcher D, Shirley S, Rodon T, Southcott C. Constraints to wildlife harvesting among aboriginal communities in Alaska and Canada. Food Secur. 2016;8(6):1153–67. Available from: <http://dx.doi.org/10.1007/s12571-016-0619-1>

18. Dombrowski K, Habecker P, Gauthier GR, Khan B, Moses J. Relocation redux: Labrador Inuit population movements and inequalities in the land claims era. Curr Anthropol. 2016;57(6):785–805. Available from: <https://doi.org/10.1086/689210>

19. Harper SL, Edge VL, Ford J, Willox AC, Wood M, McEwen SA, et al. Climate-sensitive health priorities in Nunatsiavut, Canada. BMC Public Health. 2015;15(1):1–18. Available from: <http://dx.doi.org/10.1186/s12889-015-1874-3>

20. Mushquash CJ, Stewart SH, Comeau MN, McGrath PJ. The structure of drinking motives in first nations adolescents in Nova Scotia. Am Indian Alaska Nativ Ment Heal Res. 2008;15(1):33–52. Available from: <https://doi.org/10.5820/aian.1501.2008.33>

21. Jardine CG, Boyd AD, Furgal CM. Gender and place influences on health risk perspectives in northern Canadian Aboriginal communities. Gender, Place Cult. 2009;16(2):201–23. Available from: <https://doi.org/10.1080/09663690902795837>

22. Harper SL, Edge VL, Ford J, Thomas MK, McEwen SA. Lived experience of acute gastrointestinal illness in Rigolet, Nunatsiavut: “Just suffer through it.” Soc Sci Med. 2015;126:86–98. Available from: <https://doi.org/10.1016/j.socscimed.2014.12.011>

23. Daley P, Bajgai J, Penney C, Williams K, Whitney H, Golding GR, et al. A cross sectional study of animal and human colonization with Methicillin-Resistant Staphylococcus aureus (MRSA) in an Aboriginal community. BMC Public Health. 2016;16(1):1–8. Available from: <http://dx.doi.org/10.1186/s12889-016-3220-9>

24. Latimer M, Sylliboy JR, Francis J, Amey S, Rudderham S, Finley GA, et al. Co‐creating better healthcare experiences for First Nations children and youth: The FIRST approach emerges from Two‐Eyed seeing. Paediatr Neonatal Pain. 2020;(April):1–9. Available from: <https://doi.org/10.1002/pne2.12024>

25. Durkalec A. Understanding the role of environment for Indigenous health: A case study of sea ice as a place of health and risk in the Inuit community of Nain, Nunatsiavut [Masters thesis]. Trent University; 2012.

26. Perry V. An anaylsis of social support and the health of the on-reserve Nova Scotia Mi’kmaq population [Masters thesis]. Dalhousie University; 2010

27. Latimer M, Simandl D, Finley A, Rudderham S, Harman K, Young S, et al. Understanding the impact of the pain experience on Aboriginal children’s wellbeing: Viewing through a two-eyed seeing lens. First Peoples Child Fam Rev. 2014;9(1):22–37. Available from: <https://fpcfr.com/index.php/FPCFR/article/view/183>

28. Giles M, Schiff R, Valcour J. Impact of HIV education program for youth in Southern Inuit communities. Can J Native Stud. 2017;37:49–64.

29. Durkalec A, Furgal C, Skinner MW, Sheldon T. Climate change influences on environment as a determinant of Indigenous health: Relationships to place, sea ice, and health in an Inuit community. Soc Sci Med. 2015;136–137:17–26. Available from: <http://dx.doi.org/10.1016/j.socscimed.2015.04.026>

30. Brunger F, Schiff R, Morton-Ninomiya M, Bull J. Animating the concept of “ethical space”: The Labrador Aboriginal Health Research Committee Ethics Workshop. Int J Indig Heal. 2016;10(1):3–15. Available from: <https://doi.org/10.18357/ijih.101201513194>

31. Alaghehbandan R, Sikdar KC, MacDonald D, Collins KD, Rossignol AM. Unintentional injuries among children and adolescents in Aboriginal and non-Aboriginal communities, Newfoundland and Labrador, Canada. Int J Circumpolar Health. 2010;69(1):61–71. Available from: <https://doi.org/10.3402/ijch.v69i1.17386>

32. Waldron IRG. Findings from the series of workshops “In whose backyard?—Exploring toxic legacies in Mi’kmaw and African Nova Scotian communities.” Environ Justice. 2015;8(2):33–7. Available from: <https://doi.org/10.1089/env.2014.0034>

33. Nicholson M. Press coverage of Innu youth solvent abuse: A discourse analysis of attribution of responsibility [Masters thesis]. University of Guelph; 2004

34. Caldwell D, Maloney A. It started over coffee: The Aboriginal Community Youth Resilience Network (ACYRN) in Mi’kmaq and Maliseet communities of Atlantic Canada. Pimatisiwin. 2008;6(2):129–43. Available from: <https://www.ncbi.nlm.nih.gov/pmc/articles/PMC2935487/>

35. Puiras JA. Overcoming barriers: The perspectives of Aboriginal women attending the University of Prince Edward Island [Masters thesis]. University of Prince Edward Island; 2011

36. Wilson K, Steenbeek A, Asbridge M, Cragg A, Langille DB. Sexual health among female Aboriginal university students in the Maritime Provinces of Canada: Risk behaviours and health services use. Sex Health. 2016;13(1):35–42. Available from: <https://doi.org/10.1071/SH15119>

37. Forsey RGP. Prevalence of childhood eczema and food sensitization in the First Nations reserve of Natuashish, Labrador, Canada. BMC Pediatr. 2014;14(1). Available from: <https://doi.org/10.1186/1471-2431-14-76>

38. Vernescu RM. Sustained attention training in children with fetal alcohol spectrum disorder [dissertation]. Memorial University of Newfoundland; 2008

39. Martin D, McNally M, Castleden H, Worden-Driscoll I, Clarke M, Wall D, et al. Linking Inuit knowledge and public health for improved child and youth oral health in NunatuKavut. JDR Clin Transl Res. 2018;3(3):256–63. Available from: <https://doi.org/10.1177/2380084418767833>

40. Harper SL, Edge VL, Schuster-Wallace CJ, Berke O, McEwen SA. Weather, water quality and infectious gastrointestinal illness in two Inuit communities in Nunatsiavut, Canada: Potential implications for climate change. Ecohealth. 2011;8(1):93–108. Available from: <https://doi.org/10.1007/s10393-011-0690-1>

41. Castleden H, Bennett E, Group PLNW, Lewis D, Martin D. “Put it near the Indians”: Indigenous perspectives on pulp mill contaminants in their traditional territories (Pictou Landing First Nation, Canada). Prog Community Heal Partnerships Res Educ Action. 2017;11(1):25–33. Available from: <https://doi.org/10.1353/cpr.2017.0004>

42. Brunger F, Wall D. “What do they really mean by partnerships?” Questioning the unquestionable good in ethics guidelines promoting community engagement in Indigenous health research. Qual Health Res. 2016;26(13):1862–77. Available from: <https://doi.org/10.1177/1049732316649158>

43. Laird BD, Goncharov AB, Egeland GM, Chan HM. Dietary advice on Inuit Traditional food use needs to balance benefits and risks of mercury, selenium, and n3 fatty acids. 1986;c(June 1985):8412. Available from: <https://doi.org/10.3945/jn.112.173351>

44. Dombrowski K, Khan B, Channell E, Mclean K, Misshula E. Kinship, family, and exchange in a Labrador Inuit Community. 2013;50(1):89–104. Doi: 10.3368/aa.50.1.89

45. Sawatzky A, Cunsolo A, Jones-Bitton A, Gillis D, Wood M, Flowers C, et al. “The best scientists are the people that’s out there”: Inuit-led integrated environment and health monitoring to respond to climate change in the Circumpolar North. Clim Change. 2020;160(1):45–66. Available from: <http://doi.org/10.1007/s10584-019-02647-8>

46. Mercer N, Hanrahan M. “Straight from the heavens into your bucket”: Domestic rainwater harvesting as a measure to improve water security in a subarctic indigenous community. Int J Circumpolar Health. 2017;76(1):1–9. <https://doi.org/10.1080/22423982.2017.1312223>

47. Steenbeek A, Amirault M, Saulner G, Morris C. Strengthening community-based approaches to HIV/AIDS & STI screening, treatment & prevention among Atlantic First Nation People. CJACBR*,* 2010*;*3. Available from: <https://caan.ca/wp-content/uploads/2012/05/Strengthening-Community-Based-Approaches-to-HIVAIDS-STI-Screening.pdf>

48. Sarkar A, Hanrahan M, Hudson A. Water insecurity in Canadian Indigenous communities: Some inconvenient truths. Rural Remote Health. 2015;15(4):1–13. Available from: [http://www.rrh.org.au](http://www.rrh.org.au/)

49. Wien F, Denis J, Dockstator JS, Dockstator MS, Duhaime G, Loppie C, et al. First Nation paths to well-being: lessons from the Poverty Action Research Project. Glob Health Promot. 2019;26(3_suppl):6–16. Available from: <https://doi.org/10.1177/1757975919831654>

50. Noel M, O’Connor RM, Boudreau B, Mushquash CJ, Comeau MN, Stevens D, et al. The Rutgers Alcohol Problem Index (RAPI): A comparison of cut-points in First Nations Mi’kmaq and non-Aboriginal adolescents in rural Nova Scotia. Int J Ment Health Addict. 2010;8(2):336–50. <https://doi.org/10.1007/s11469-009-9219-z>

51. Pace J, Gabel C. Using photovoice to understand barriers and enablers to Southern Labrador Inuit intergenerational interaction: Research. J Intergener Relatsh. 2018;16(4):351–73. Available from: <https://doi.org/10.1080/15350770.2018.1500506>

52. Mioc D, Anton F, Ahmad A. Mapping online the environmental impact of mining operations in New Brunswick. WIT Trans Ecol Environ. 2015;199(1):135–43. <https://doi.org/10.2495/RAV150121>

53. Vukic A, Jesty C, Mathews SV, Etowa J. Understanding race and racism in nursing: Insights from Aboriginal nurses. ISRN Nurs. 2012;2012:1–9. <https://doi.org/10.5402/2012/196437>

54. Liebenberg L, Wall D, Wood M, Hutt-MacLeod D. Spaces & places: Understanding sense of belonging and cultural engagement among Indigenous youth. Int J Qual Methods [Internet]. 2019;18:1–10. Available from: <https://doi.org/10.1177/1609406919840547>

55. Cunsolo Willox A, Harper SL, Edge VL. Storytelling in a digital age: Digital storytelling as an emerging narrative method for preserving and promoting indigenous oral wisdom. Qual Res. 2013;13(2):127–47. <https://doi.org/10.1177/1468794112446105>

56. Edwards N, Alaghehbandan R, MacDonald D, Sikdar K, Collins K, Avis S. Suicide in Newfoundland and Labrador: A linkage study using medical examiner and vital statistics data. Can J Psychiatry. 2008;53(4):252–9. Available from: <https://doi.org/10.1177/070674370805300406>

57. Alaghehbandan R, Sikdar KC, Gladney N, MacDonald D, Collins KD. Epidemiology of severe burn among children in Newfoundland and Labrador, Canada. Burns [Internet]. 2012;38(1):136–40. Available from: <http://dx.doi.org/10.1016/j.burns.2011.06.010>

58. Hanrahan M, Sarkar A, Hudson A. Exploring water insecurity in a northern indigenous community in Canada: The “never-ending job” of the Southern Inuit of Black Tickle, Labrador. Arctic Anthropol. 2014;51(2):9–22. Available from: <https://doi.org/10.3368/aa.51.2.9>

59. Pace J. “Place-ing” dementia prevention and care in NunatuKavut, Labrador. Can J Aging. 2020;39(2):247–62.

60. Thompson S. Environmental justice in a toxic economy: Community struggles with environmental health disorders in Nova Scotia [dissertation]. University of Toronto; 2002

61. Harris S. The role of ecotourism in Aboriginal community development: The case of Lennox Island First Nation [Masters thesis]. Acadia University; 2005

62. Webb MJ. An assessment and review of currently existing databases as a foundation for the monitoring of health status of a population within a defined geographic location [Masters Thesis]. Memorial University of Newfoundland; 2001

63. Berghout J, Miller JD, Mazerolle R, O’Neill L, Wakelin C, Mackinnon B, et al. Indoor environmental quality in homes of asthmatic children on the Elsipogtog Reserve (NB), Canada. Int J Circumpolar Health. 2005;64(1):77–85. Available from: <https://doi.org/10.3402/ijch.v64i1.17956>

64. Blackstock C. When Everything Matters: Comparing the experiences of first nations and non-aboriginal children removed from their families in Nova Scotia from 2003 to 2005 [dissertation]. University of Toronto; 2009

65. Taylor JP, Timmons V, Larsen R, Walton F, Bryanton J, Critchley K, et al. Nutritional concerns in Aboriginal children are similar to those in Non-Aboriginal children in Prince Edward Island, Canada. J Am Diet Assoc. 2007;107(6):951–5. Available from: <https://doi.org/10.1016/j.jada.2007.03.008>

66. Spillane NS, Cyders MA, Maurelli K. Negative urgency, problem drinking and negative alcohol expectancies among members from one First Nation: A moderated-mediation model. Addict Behav. 2012;37(11):1285–8. Available from: <http://dx.doi.org/10.1016/j.addbeh.2012.06.007>

67. Latimer M, Sylliboy JR, MacLeod E, Rudderham S, Francis J, Hutt-MacLeod D, et al. Creating a safe space for First Nations youth to share their pain. Pain Reports. 2018;3(7):1–12. Available from: <https://doi.org/10.1097/PR9.0000000000000682>

68. Brass GM, Gordon M, Bernard B. Invited commentary: ACCESS Open Minds National Indigenous Council. Early Interv Psychiatry. 2019;13(S1):71–3. Available [from: https://doi.org/10.1111/eip.12822](from:%20https://doi.org/10.1111/eip.12822)

69. Kovesi T. Respiratory medicine in Nunavut and Northern Canada. Can J Respir Crit Care, Sleep Med. 2019;0(0):16. Available from: <https://doi.org/10.1080/24745332.2018.1483784>

70. Reeves AJ. Honouring womanhood: Understanding the conceptualization and social construction of young adult First Nation women's sexuality in Atlantic Canada [Masters thesis]. Dalhousie University; 2008

71. Gibson K, O’Donnell S, Coulson H, Kakepetum-Schultz T. Mental health professionals’ perspectives of telemental health with remote and rural first nations communities. J Telemed Telecare. 2011;17(5):263–7. Available from: <https://doi.org/10.1258/jtt.2011.101011>

72. Whitty-Rogers J, Caine V, Cameron B. Aboriginal womenʼs experiences with gestational diabetes mellitus. Adv Nurs Sci. 2016;39(2):181–98. Available from: <https://doi.org/10.1097/ans.0000000000000115>

73. Ford JD, Bolton KC, Shirley J, Pearce T, Tremblay M, Westlake M. Research on the human dimensions of climate change in Nunavut, Nunavik, and Nunatsiavut: A literature review and gap analysis. Arctic. 2012;65(3):289–304. Available from: <https://www.jstor.org/stable/41758936>

74. Baker C, Manju V, Tanaka C. Sticks and Stone: Racism as experienced by adolescents in New Brunswick. Can J Nurs Res. 2001;33(3):87–105. Available from: <https://cjnr.archive.mcgill.ca/article/view/1650/1650>

75. Atikessé L, De Grosbois SB, St-Jean M, Penashue B, Benuen M. Innu food consumption patterns: Traditional food and body mass index. Can J Diet Pract Res. 2010;71(3):41–50. Available from: <https://doi.org/10.3148/71.3.2010.125>

76. King N, Vriezen R, Edge VL, Ford J, Wood M, Harper S. The hidden costs: Identification of indirect costs associated with acute gastrointestinal illness in an Inuit community. PLoS One. 2018;13(5):1–22. Available from: <http://dx.doi.org/10.1371/journal.pone.0196990>

77. Hachey S, Clovis J, Lamarche K. Children’s oral health and barriers to seeking care: Perspectives of caregivers seeking pediatric hospital dental treatment. Health Policy. 2019;15(1):29–39. Available from: [10.12927/hcpol.2019.25940](file:///C:\Users\Kathleen\Downloads\10.12927\hcpol.2019.25940)

78. Calder RSD, Bromage S, Sunderland EM. Risk tradeoffs associated with traditional food advisories for Labrador Inuit. Environ Res. 2019;168(September 2018):496–506. Available from: <https://doi.org/10.1016/j.envres.2018.09.005>

79. Sarkar A. Environmental impact assessment of uranium mining on Indigenous land in Labrador (Canada): Biases and manipulations. Environ Justice. 2019;12(2):61–8. Available from: <https://doi.org/10.1089/env.2018.0036>

80. MacDonald J, Harper SL, Cunsolo Willox A, Edge VL. A necessary voice: Climate change nd lived experiences of youth in Rigolet, Nunatsiavut, Canada. Glob Environ Chang. 2013;23(1):360–71. Available from: <http://dx.doi.org/10.1016/j.gloenvcha.2012.07.010>

81. Moore C, Castleden HE, Tirone S, Martin D. Implementing the Tri-Council Policy on Ethical Research involving Indigenous Peoples in Canada: So, how’s that going in Mi’kma’ki? Int Indig Policy J. 2017;8(2). Available from: <https://doi.org/10.18584/iipj.2017.8.2.4>

82. Richards G, Frehs J, Myers E, Van Bibber M. The climate change and health adaptation program: Indigenous climate Leaders’ championing adaptation efforts. Heal Promot Chronic Dis Prev Canada. 2019;39(4):127–30. Available from: <https://www.ncbi.nlm.nih.gov/pmc/articles/PMC6553577/>

83. Jacono J, Jacono B. The use of puppetry for health promotion and suicide prevention among Mi’Kmaq youth. J Holist Nurs. 2008;26(1):50–5. Available from: [https://doi.org/10.1177/0898010107306201](https://doi.org/10.1177%2F0898010107306201)

84. McIntyre L, Wien F, Rudderham S, Etter L, Moore C, MacDonald N, et al. A gender analysis of the stress experience of young Mi’kmaq women. Res Bull Centres Excell Women’s Heal. 2003;4(1):7–10. Available from: <http://www.cwhn.ca/sites/default/files/PDF/CEWH/RB/bulletin-vol4no1EN.pdf>

85. Coumans C. Research on contested ground: Women, mining and health. Pimatisiwin A J Aborig Indig Community Heal. 2005;3(1):9–32. Available from: <http://www.pimatisiwin.com/uploads/426573685.pdf>

86. Martin DH. “Now we got lots to eat and they’re telling us not to eat it”: understanding changes to south-east Labrador Inuit relationships to food. Int J Circumpolar Health. 2011;70(4):384–5. Available from: <https://doi.org/10.3402/ijch.v70i4.17842>

87. Harper SL, Edge VL, Ford J, Thomas MK, Pearl DL, Shirley J, et al. Acute gastrointestinal illness in two Inuit communities: Burden of illness in Rigolet and Iqaluit, Canada. Epidemiol Infect. 2015;143(14):3048–63. Available from: <https://doi.org/10.1017/S0950268814003744>

88. Schiff R, Brunger F. Northern food networks: Building collaborative efforts for food security in remote Canadian Aboriginal communities. J Agric Food Syst Community Dev. 2013;3(3):121–38. Available from: <https://doi.org/10.5304/jafscd.2013.033.012>

89. Bull J, Hudson A. Research governance in NunatuKavut: engagement, expectations and evolution. Int J Circumpolar Health. 2018;77(1):0–3. Available from: <https://doi.org/10.1080/22423982.2018.1556558>

90. Whitty-Rogers J. Childbirth experiences of women form one Mi’kmaq community in Nova Scotia [Masters Thesis]. Dalhousie University; 2006

91. Ungar M, Brown M, Liebenberg L, Cheung M, Levine K. Distinguishing differences in pathways to resilience among Canadian youth. Can J Community Ment Heal [Internet]. 2008;27(1):1–13. Available from: <http://cjcmh.com/doi/10.7870/cjcmh-2008-0001>

92. Morton Ninomiya ME, Hurley N, Penashue J. A decolonizing method of inquiry: using institutional ethnography to facilitate community-based research and knowledge translation. Crit Public Health. 2020;30(2):220–31. Available from: <https://doi.org/10.1080/09581596.2018.1541228>

93. Webster D, Weerasinghe S, Stevens P. Morbidity and mortality rates in a Nova Scotia First Nations community, 1996-1999. Can J Public Heal. 2004;95(5):369–75. Available from: www.jstor.org/stable/41994404

94. Macdonald C, Martin-misener R, Steenbeek A, Browne A, Macdonald C, Martin-misener R, et al. Honouring stories: Mi’kmaq women’s experiences with pap screening in Eastern Canada. 2015;47:72–96. Available from: [10.1177/084456211504700106](https://pubmed.ncbi.nlm.nih.gov/29509451/)

95. Li YC. Modeling the Inuit diet to minimize contaminant while maintaining nutrient intakes [Masters thesis]. McGill University; 2007

96. Vukic A, Gregory D, Martin-Misener R, Etowa J. Aboriginal and western conceptions of mental health and lllness. Pimatisiwin A J Aborig Indig Community Heal. 2011;9(1):65–86. Available form: <https://journalindigenouswellbeing.com/media/2018/12/4_Vukic.pdf>

97. Kenny TA, Fillion M, Simpkin S, Wesche SD, Chan HM. Caribou (Rangifer tarandus) and Inuit nutrition security in Canada. Ecohealth. 2018;15(3):590–607. Available from: <https://doi.org/10.1007/s10393-018-1348-z>

98. Davidson K, Holderby A, Willis S, Barksdale C, Richardson T, Loppie C, et al. Three top Canadian and personal health concerns of a random sample of Nova Scotia women. Can J Public Heal. 2001;92(1):53–6. Available from: <https://doi.org/10.1007/BF03404845>

99. Petrasek MacDonald J, Cunsolo Willox A, Ford JD, Shiwak I, Wood M, Wolfrey C, et al. Protective factors for mental health and well-being in a changing climate: Perspectives from Inuit youth in Nunatsiavut, Labrador. Soc Sci Med. 2015;141:133–41. Available from: <https://doi.org/10.1016/j.socscimed.2015.07.017>

100. Ostapchuk J, Harper S, Cunsolo Willox A, Edge VL, Community Government RI. Exploring Elders’ and Seniors’ Perceptions of How Climate Change is Impacting Health and Well-being in Rigolet, Nunatsiavut. Int J Indig Heal. 2017;9(2):6. Available from: <https://doi.org/10.18357/ijih92201214358>

101. Brazier B. Intimate partner abuse: First Nations women’s experience [dissertation]. University of New Brunswick; 2006

102. Tatemichi S, Miedema B, Leighton S. Breast cancer screening First Nations communities in New Brunswick. Can Fam Physician. 2002;38. Available from: <https://doi.org/10.1017/pls.2018.3>

103. Severini A, Jiang Y, Brassard P, Morrison H, Demers AA, Oguntuase E, et al. Type-specific prevalence of human papillomavirus in women screened for cervical cancer in Labrador, Canada. Int J Circumpolar Health. 2013;72(1):0–5. Available from: <https://doi.org/10.3402/ijch.v72i0.19743>

104. Lavoie JG. Policy silences: Why Canada needs a national first nations, Inuit and Métis health policy. Int J Circumpolar Health. 2013;72(1):1–7. Available from: <https://doi.org/10.3402/ijch.v72i0.22690>

105. Rosol R, Powell-Hellyer S, Chan LHM. Impacts of decline harvest of country food on nutrient intake among Inuit in Arctic Canada: Impact of climate change and possible adaptation plan. Int J Circumpolar Health. 2016;75. Available from: <https://doi.org/10.3402/ijch.v75.31127>

106. MacDonald JP, Ford J, Willox AC, Mitchell C, Productions K. Youth-led participatory video as a strategy to enhance Inuit youth adaptive capacities for dealing with climate change. Arctic. 2015;68(4):486–99. Available from: <https://doi.org/10.14430/arctic4527>

107. Latimer M, Rudderham S, Lethbridge L, MacLeod E, Harman K, Sylliboy JR, et al. Occurrence of and referral to specialists for pain-related diagnoses in First Nations and non-First Nations children and youth. CMAJ. 2018;190(49):E1434–40. Available from: <https://doi.org/10.1503/cmaj.180198>

108. Durkalec A, Furgal C, Skinner MW, Sheldon T. Investigating environmental determinants of injury and trauma in the Canadian north. Int J Environ Res Public Health. 2014;11(2):1536–48. Available from: <https://doi.org/10.3390/ijerph110201536>

109. Hutt-MacLeod D, Rudderham H, Sylliboy A, Sylliboy-Denny M, Liebenberg L, Denny JF, et al. Eskasoni First Nation’s transformation of youth mental healthcare: Partnership between a Mi’kmaq community and the ACCESS Open Minds research project in implementing innovative practice and service evaluation. Early Interv Psychiatry. 2019;13(S1):42–7. Available from: https://doi.org/10.1111/eip.12817

110. Spillane NS, Smith GT. Individual differences in problem drinking among tribal members from one first nation community. Alcohol Clin Exp Res. 2010;34(11):1985–92. Available form: <https://doi.org/10.1111/j.1530-0277.2010.01288.x>

111. Cunsolo Willox A, Harper SL, Edge VL, Landman K, Houle K, Ford JD. The land enriches the soul: On climatic and environmental change, affect, and emotional health and well-being in Rigolet, Nunatsiavut, Canada. Emot Sp Soc. 2013;6(1):14–24. Available from: <https://doi.org/10.1016/j.emospa.2011.08.005>

112. Harper SL, Edge VL, Schuster-Wallace CJ, Ar-Rushdi M, McEwen SA. Improving Aboriginal health data capture: Evidence from a health registry evaluation. Epidemiol Infect. 2011;139(11):1774–83. Available from: <https://doi.org/10.1017/S095026881000275X>

113. Etowa J, Jesty C, Vukic A. Indigenous nurses’ stories: Perspectives on the cultural context of aboriginal health care work. Can J Native Stud. 2011;31(2):29–46.

114. Wilkins R, Uppal S, Finès P, Senècal S, Guimond E, Dion R. Life expectancy in the Inuit-inhabited areas of Canada, 1989 to 2003. Health Rep. 2008;19(1):7–19. Available from: <https://www150.statcan.gc.ca/n1/en/pub/82-003-x/2008001/article/10463-eng.pdf?st=nyZ3UfVF>

115. Goldhar, C. Water ways: Vulnerability to freshwater changes in the Inuit settlement region of Nunatsiavut, Labrador [Masters thesis]. Memorial University of Newfoundland; 2011

116. Caldwell D. The Suicide Prevention Continuum. Pimatisiwin. 2008;6(2):145–53. Available from: <https://www.ncbi.nlm.nih.gov/pmc/articles/PMC2936581/>

117. Jardine C, Furgal C. Knowledge translation with northern Aboriginal communities: A case study. Can J Nurs Res. 2010;42(1):119–27. Available from: <https://cjnr.archive.mcgill.ca/article/view/2239/2233>

118. Martin D, McNally M, Castleden H, Bornstien S, Clovis J, Filiaggi M. Kungatsiajuk (Healthy Smiles) project research team report on child and youth oral health survey results for NunatuKavut, 2012–13. *Network for Canadian Oral Health Research.* 2016. Available from: <https://kungatsiajuk.ca/wp-content/uploads/2013/04/NCOHR_Kungatsiajuk_final_1.pdf>

119. Mushquash CJ, Stewart SH, Mushquash AR, Comeau MN, McGrath PJ. Personality traits and drinking motives predict alcohol misuse among Canadian Aboriginal youth. Int J Ment Health Addict. 2014;12(3):270–82. Available from: <https://doi.org/10.1007/s11469-013-9451-4>

120. Hanrahan M, Sarkar A, Hudson A. Water insecurity in Indigenous Canada: A case study of illness, neglect, and urgency. Int Conf Mar Freshw Environ iMFE 2014. 2014;1–9. Available from: <http://nlwater.ruralresilience.ca/wp-content/uploads/2014/09/iMFE-Paper-Submission-Revised-August-2014.pdf>

121. Giles BG. Exploring Aboriginal views of health using fuzzy cognitive maps and transitive closure. 2008;99(5):411–7. Available from: <https://doi.org/10.1007/BF03405252>

122. Pufall E. Engaging northern communities in monitoring traditional country foods for zoonotic anisakid nematodes [Masters thesis]. University of Guelph; 2010

123. Oberndorfer E, Winters N, Gear C, Ljubicic G, Lundholm J. Plants in a “sea of relationships”: Networks of plants and fishing in Makkovik, Nunatsiavut (Labrador, Canada). J Ethnobiol. 2017;37(3):458–77. Available from: <https://doi.org/10.2993/0278-0771-37.3.458>

124. Loppie C. Grandmothers’ voices: Mi’kmaq women and menopause [dissertation]. Dalhousie University; 2004

125. Brunger F, Russell T. Risk and representation in research ethics: The NunatuKavut experience. J Empir Res Hum Res Ethics. 2015;10(4):368–79. Available from: <https://doi.org/10.1177/1556264615599687>

126. Hanrahan MC. Identifying the needs of Innu and Inuit patients in urban health settings in Newfoundland and Labrador. Can J Public Heal. 2002;93(3):149–52. Available from: <https://doi.org/10.1007/BF03404558>

127. Cunsolo Willox A, Harper SL, Ford JD, Landman K, Houle K, Edge VL. “From this place and of this place:” Climate change, sense of place, and health in Nunatsiavut, Canada. Soc Sci Med. 2013;75(3):538–47. Available from: <https://doi.org/10.1007/s10584-013-0875-4>

128. Samson C, Pretty J. Environmental and health benefits of hunting lifestyles and diets for the Innu of Labrador. Food Policy. 2006;31(6):528–53. Available from: <https://doi.org/10.1016/j.foodpol.2006.02.001>

129. Kipp A, Cunsolo A, Gillis D, Sawatzky A, Harper SL. The need for community-led, integrated and innovative monitoring programmes when responding to the health impacts of climate change. Int J Circumpolar Health. 2019;78(2). Available from: <https://doi.org/10.1080/22423982.2018.1517581>

130. Cunsolo Willox A, Harper SL, Ford JD, Edge VL, Landman K, Houle K, et al. Climate change and mental health: An exploratory case study from Rigolet, Nunatsiavut, Canada. Clim Change. 2013;121(2):255–70. Available from: <https://doi.org/10.1007/s10584-013-0875-4>

131. Nudell Z. Colonization embodied: Diabetes in Sheshatshiu [Masters thesis]. Saint Mary’s University; 2006.

132. Owusu-Bempah A, Kanters S, Druyts E, Toor K, Muldoon KA, Farquhar JW, et al. Years of life lost to incarceration: Inequities between Aboriginal and non-Aboriginal Canadians. BMC Public Health. 2014;14(1):1–6. Available from: <https://doi.org/10.1186/1471-2458-14-585>

133. Anderson D, Ford JD, Way RG. The impacts of climate and social changes on Cloudberry (Bakeapple) picking: A case study from Southeastern Labrador. Hum Ecol. 2018;46(6):849–63. Available from: <https://doi.org/10.1007/s10745-018-0038-3>

134. Hanrahan M, Mercer N. Gender and water insecurity in a subarctic Indigenous community. Can Geogr. 2018;(xx):1–14. Available from: <https://doi.org/10.1111/cag.12508>

135. Pufall EL, Jones AQ, Mcewen SA, Lyall C, Peregrine AS, Edge VL. Perception of the importance of traditional country foods to the physical , mental , and spiritual health of Labrador Inuit content in a trusted digital archive. 2011;64(2):242–50. Available from: <https://doi.org/10.14430/arctic4103>

136. Huet C, Rosol R, Egeland GM. The prevalence of food insecurity is high and the diet quality poor in Inuit communities. J Nutr. 2012;142(3):541–7. Available from: <https://doi.org/10.3945/jn.111.149278>

137. Vukic A, Rudderham S, Martin-Misener R. A community partnership to explore gaps in mental health services in First Nations communities in Nova Scotia. Can J Public Heal. 2009;100(6):432–5. Available from: <https://doi.org/10.1007/BF03404339>

138. Brunger F, Bull J. Whose agenda is it? Regulating health research ethics in LabradorWhose agenda is it? Regulating health research ethics in Labrador. Études/Inuit/Studies. 2012;35(1–2):127. Available from: <https://doi.org/10.7202/1012838ar>

139. Tarasuk V, Fafard St-Germain AA, Mitchell A. Geographic and socio-demographic predictors of household food insecurity in Canada, 2011-12. BMC Public Health. 2019;19(1):1–12. Available from: <https://doi.org/10.1186/s12889-018-6344-2>

140. Larmer JR. The highway runs east: Poverty, policing, and the missing and murdered Indigenous women of Nova Scotia. Dalhousie J Leg Stud. 2018;27:89–136.

141. Zhou YE, Kubow S, Egeland GM. Highly unsaturated n-3 fatty acids status of Canadian Inuit: International Polar Year Inuit Health Survey, 2007–2008. Int J Circumpolar Health. 2011;70(5):498–510. Available from: <https://doi.org/10.3402/ijch.v70i5.17864>

142. Lane J, Carrier L, Jefferies K, Yu Z. Diverse representation in nursing leadership: Developing a shared position statement on allyship. Creat Nurs. 2019;25(4):316-321.

143. Peddle KM. Youth Culture - Say what? Negotiating rural space in daily life in Southern Labrador: A Participatory Photovoice Project [dissertation]. Conordia University; 2008.

144. Critchley K, Walton F, Timmons V, Bryanton J, McCarthy M J, Taylor J. Personal health practices around physical activity as perceived by the Aboriginal children of Prince Edward Island. J Aborig Health. 2006;3(1):26-33. Available from: <https://doi.org/10.3138/ijih.v3i1.28952>

145. El Hayek Fares J, Weiler HA. Vitamin D status and intake of lactating Inuit women living in the Canadian Arctic. Public Health Nutr. 2018;21(11):1988–94. Available from: <https://doi.org/10.1017/S1368980017004189>

146. Spillane NS, Greenfield B, Venner K, Kahler CW. Alcohol use among reserve-dwelling adult First Nation members: Use, problems, and intention to change drinking behavior. Addict Behav. 2015;41:232–7. Available from: <https://doi.org/10.1016/j.addbeh.2014.10.015>

147. Loppie C. Learning from the grandmothers: incorporating Indigenous principles into qualitative research. Qual Health Res. 2007;17(2):276–84. Available from: <https://doi.org/10.1177/1049732306297905>

148. Hanrahan M, Sarkar A, Hudson A. Water insecurity in Indigenous Canada: A community-based inter-disciplinary approach. Water Qual Res J Canada. 2016;51(3):270–81. Available from: <https://doi.org/10.2166/wqrjc.2015.010>

149. Langan LA, Caissie R. Occurrence of otitis media and hearing loss among First Nations elementary school children Occurrence de l’otite moyenne et de la perte auditive chez les écoliers des Premières Nations. Audiology. 2007;31(4):178–85. Available from: <https://cjslpa.ca/files/2007_CJSLPA_Vol_31/No_04_161-200/Langan_Sockalingam_CJSLPA_2007.pdf>

150. Getty GA. The journey between Western and Indigenous research paradigms. J Transcult Nurs. 2010;21(1):5–14. Available from: <https://doi.org/10.1177/1043659609349062>

151. Larsen, R. Food and health perceptions of Mi’kmaq children and youth in Prince Edward Island [Masters thesis]. University of Prince Edward Island; 2005.

152. Organ J, Castleden H, Furgal C, Sheldon T, Hart C. Contemporary programs in support of traditional ways: Inuit perspectives on community freezers as a mechanism to alleviate pressures of wild food access in Nain, Nunatsiavut. Heal Place. 2014;30:251–9. Available from: <http://dx.doi.org/10.1016/j.healthplace.2014.09.012>

153. Pictou Landing. Community food security in Pictou Landing. 2014;1–30. Available from: <https://foodarc.ca/wp-content/uploads/2014/05/CFS-in-PLFN-project-report-UPDATED-July-2014.pdf>

154. Alwyn E. Traditions in a colonized world: Two realities of a First Nation [dissertation]. University of Toronto; 2004.

155. Laird BD, Goncharov AB, Chan HM. Body burden of metals and persistent organic pollutants among Inuit in the Canadian Arctic. Environ Int. 2013;59:33–40. Available from: <https://doi.org/10.1016/j.envint.2013.05.010>

156. Bull JR. Research with Aboriginal Peoples: Authentic relationships as a precursor to ethical research. J Empir Res Hum Res Ethics. 2010;5(4):13–22. Available from: <https://doi.org/10.1525/jer.2010.5.4.13>

157. Goldhar C, Bell T, Wolf J. Rethinking existing approaches to water security in remote communities: An analysis of two drinking water systems in Nunatsiavut, Labrador, Canada. Water Alternatives*,* 2013;6(3):462-86. Available from: <https://dlc.dlib.indiana.edu/dlc/bitstream/handle/10535/9241/Art6-3-8.pdf?sequence=1&isAllowed=y>

158. Pollock NJ, Mulay S, Valcour J, Jong M. Suicide rates in aboriginal communities in Labrador, Canada. Am J Public Health. 2016;106(7):1309–15. Available from: <https://doi.org/10.2105/AJPH.2016.303151>

159. Batal M, Decelles S. A scoping review of obesity among Indigenous Peoples in Canada. J Obes. 2019;2019. Available from: <https://doi.org/10.1155/2019/9741090>

160. Alaghehbandan R, Gates KD, MacDonald D. Hospitalization due to pneumonia among Innu, Inuit and non-Aboriginal communities, Newfoundland and Labrador, Canada. Int J Infect Dis. 2007;11(1):23–8. Available from: <https://doi.org/10.1016/j.ijid.2005.09.003>

161. Dean LS. Environment and health risk communication pathways in Aboriginal communities: Learning from the case of foodweb contaminants and nutrition issues with young Inuit women in Nunatsiavut [Masters thesis]. Dalhousie University; 2009.

162. Zahradnick M, Stewart S, Stevens D, Wekerle C. Knowledge translation in a community-based study of the relations among violence exposure, post-traumatic stress, and alcohol misuse in M’kmaq youth. First Peoples Child Fam Rev. 2009;4:106–17. Available from: <https://fpcfr.com/index.php/FPCFR/article/view/142/125>

163. Beaton M. Selling tragedy to an indifferent audience: The failure of the Labrador Innu social problem [Masters thesis]. Queen’s University; 2003.

164. Polland D. The political rhetoric of social problems: Gasoline sniffing among the Innu of Labrador [Masters thesis]. Memorial University of Newfoundland; 2006.

165. Paterson BL, Sock LA, LeBlanc D, Brewer J. Ripples in the water: a toolkit for Aboriginal people on hemodialysis. CANNT J. 2010;20(1):20–8.

166. Gabel C, Pace J, Ryan C. Using photovoice to understand intergenerational influences on health and well-being in a Southern Labrador Inuit Ccmmunity. Int J Indig Heal. 2016;11(1):75. Available from: <https://doi.org/10.18357/ijih111201616014>

167. Mushquash C. Invited commentary: Community adaptations to ACCESS Open Minds—Lessons from Eskasoni and Ulukhaktok. Early Interv Psychiatry. 2019;13(S1):78–80. Available from: <https://doi.org/10.1111/eip.12825>

168. Woodman TA. Doing the best we can: Barriers and supports to healthy choices during pregnancy among Aboriginal women in Nova Scotia [Masters thesis]. Dalhousie University; 2006

169. Jamieson JA, Weiler HA, Kuhnlein H V., Egeland GM. Prevalence of unexplained anaemia in Inuit men and Inuit post-menopausal women in Northern Labrador: International polar year inuit health survey. Can J Public Heal. 2016;107(1):e81–7. Available from: <https://doi.org/10.17269/CJPH.107.5173>

170. Bowers, R. Factors affecting participation in a diabetes prevention and treatment program [Masters thesis]. Mount Saint Vincent University; 2007

171. Zahradnik M, Stewart SH, O’Connor RM, Stevens D, Ungar M, Wekerle C. Resilience moderates the relationship between exposure to violence and posttraumatic reexperiencing in Mi’kmaq youth. Int J Ment Health Addict. 2010;8(2):408–20. Available from: <https://doi.org/10.1007/s11469-009-9228-y>

172. Hackett C, Furgal C, Angnatok D, Sheldon T, Karpik S, Baikie D, et al. Going off, Growing strong: Building resilience of Indigenous youth. Can J Community Ment Heal. 2016;35(2):79–82. Available from: <https://doi.org/10.7870/cjcmh-2016-028>

173. Sarkar A, Wilton DH, Fitzgerald E. Indoor radon in micro-geological setting of an indigenous community in Canada: A pilot study for hazard identification. Int J Occup Environ Med. 2017;8(2):69–79. Available from: <https://doi.org/10.15171/ijoem.2017.1001>

174. Goyette S, Cao Z, Libman M, Ndao M, Ward BJ. Seroprevalence of parasitic zoonoses and their relationship with social factors among the Canadian Inuit in Arctic regions. Diagn Microbiol Infect Dis. 2014;78(4):404–10. Available from: <http://dx.doi.org/10.1016/j.diagmicrobio.2013.08.026>

175. Loppie C. Grandmothers’ voices: Mi’kmaq women’s vision of mid-life change. Pimatisiwin. 2005;3(2). Available from: <https://journalindigenouswellbeing.com/media/2018/10/3_Loppie.pdf>

176. Owens SL. Climate change and health: A project with women in Labrador. North. [Masters thesis]. Laval University; 2002

177. Lewis D, Castleden H, Francis S, Strickland K, Denny C. Increasing response rates on face-to-face surveys with Indigenous communities in Canada: Lessons from Pictou Landing. Prog Community Heal Partnerships Res Educ Action. 2016;10(2):197–205. Available from: <https://doi.org/10.1353/cpr.2016.0021>

178. Molema A. Errors of commission: Canada’s legacy of Indian Residential Schools [dissertation]. University of Toronto; 2016

179. Saini M. Participatory methods for Inuit public health promotion and programme evaluation in Nunatsiavut, Canada [Masters thesis]. University of Guelph; 2017

180. Moulton D. Suicide rate higher for indigenous people in Labrador. CMAJ. 2016;188(12):E275. Available from: <https://doi.org/10.1503/cmaj.109-5305>

181. Reid B. Positionality and research: “Two-Eyed Seeing” with a rural Ktaqmkuk Mi’kmaw community. Int J Qual Methods. 2020;19:1–12. Available from: <https://doi.org/10.1177/1609406920910841>

182. Wilson E. Exploring and evaluating personal, cultural and social food needs and the role of a community freezer among Inuit in Hopedale, Nunatsiavut [Masters thesis]. Trent University; 2016

183. Latimer M, Finley GA, Rudderham S, Inglis S, Francis J, Young S, et al. Expression of pain among Mi’kmaq children in one Atlantic Canadian community: a qualitative study. C Open. 2014;2(3):E133–8. Available from: <https://doi.org/10.9778/cmajo.20130086>

184. Mushquash CJ, Comeau MN, Stewart SH. An alcohol abuse early intervention approach with Mi’kmaq adolescents. First Peoples Child Fam Rev. 2007;3:17–26. Available from: <https://fpcfr.com/index.php/FPCFR/article/view/34>

185. Alaghehbandan R, Gates KD, MacDonald D. Suicide attempts and associated factors in Newfoundland and Labrador, 1998-2000. Can J Psychiatry. 2005;50(12):762–8. Available from: <https://doi.org/10.1177/070674370505001205>

186. Mendez I, Jong M, Keays-White D, Turner G. The use of remote presence for health care delivery in a northern Inuit community: A feasibility study. Int J Circumpolar Health. 2013;72(SUPPL.1):1–8. Available from: <https://doi.org/10.3402/ijch.v72i0.21112>

187. Gallant V, Duvvuri V, McGuire M. Tuberculosis in Canada - Summary 2015. Canada Commun Dis Rep. 2017;43(3/4):77–82. Available from: <https://doi.org/10.14745/ccdr.v43i34a04>

188. Harper SL, Edge VL, Cunsolo Willox A. “Changing climate, changing health, changing stories” profile: Using an EcoHealth approach to explore impacts of climate change on Inuit health. Ecohealth. 2012;9(1):89–101. Available from: <https://doi.org/10.1016/j.socscimed.2012.03.043>

189. Martin DH. The changing nature of social relations: young women’s perceptions of their health and health-related practices in three coastal Newfoundland and Labrador communities [Masters thesis]. Dalhousie University; 2004

190. Burns L, Whitty-Rogers J, MacDonald C. Understanding Mi’kmaq women’s experiences accessing prenatal care in rural Nova Scotia. ANS Adv Nurs Sci. 2019;42(2):139–55. Available from: <https://doi.org/10.1097/ANS.0000000000000248>

191. Middleton J, Cunsolo A, Jones-Bitton A, Wright CJ, Harper SL. Indigenous mental health in a changing climate: a systematic scoping review of the global literature. Environ Res Lett. 2020;15(5):053001. Available from: https://doi.org/ 10.1088/1748-9326/ab68a9

192. Dombrowski K, Channell E, Khan B, Moses J, Misshula E. Out on the land: Income, subsistence activities, and food sharing networks in Nain, Labrador. J Anthropol. 2013;2013:1–11. Available from: <https://doi.org/10.1155/2013/185048>

193. Zahradnik M, Stevens D, Stewart S, Comeau MN. Building a collaborative understanding of pathways to adolescent alcohol misuse in a Mi’kmaq community: A process paper. Practice. 2007;3(2):27–36. Available from: <https://fpcfr.com/index.php/FPCFR/article/view/37>

194. Castleden H, Watson R, Partnership T, Bennett E, Masuda J, King M, et al. Asthma prevention and management for Aboriginal people: Lessons from Mi’kmaq. 2016;1–9. Available from: <http://dx.doi.org/10.5888/pcd13.15024>

195. Landry M, Veilleux N, Arseneault J-E, Abboud S, Barrieau A, Bélanger M. Impact of a methadone maintenance program on an Aboriginal community: a qualitative study: Table 1: C Open. 2016;4(3):E431–5. Available from: <https://doi.org/10.9778/cmajo.20150076>

196. Pollock N, Cunsolo A. Collaborative approaches to wellness and health equity in the Circumpolar North: Introduction to the Special Issue. Int J Circumpolar Health [Internet]. 2019;78(2). Available from: <https://doi.org/10.1080/22423982.2019.1608084>

197. Minich K, Saudny H, Lennie C, Wood M, Williamson-Bathory L, Cao Z, et al. Inuit housing and homelessness: results from the International Polar Year Inuit Health Survey 2007–2008. Int J Circumpolar Health. 2011;70(5):520–31. Available from: <https://doi.org/10.3402/ijch.v70i5.17858>

198. Hirsch R, Furgal C, Hackett C, Sheldon T, Bell T, Angnatok D, et al. Going off, growing strong: A program to enhance individual youth and community resilience in the face of change in Nain. 2016;40(1):63. Available from: <https://doi.org/10.7202/1040145ar>

199. Iwama M, Marshall M, Marshall A, Bartlett C. Two-Eyed Seeing and the language of healing in community-based research. Can J Nativ Educ. 2009;32(2):3–23. Available from: <https://doi.org/10.1037/trm0000067>

200. Harper SL, Berrang-Ford L, Carcamo C, Llanos A, Lwasa S, Namanya DB, et al. Healthcare use for acute gastrointestinal illness in two Inuit communities: Rigolet and Iqaluit, Canada. Int J Circumpolar Health. 2015;74(May). Available from: <https://doi.org/10.3402/ijch.v74.26290>

201. Moore S. Language and identity in an Indigenous teacher education program. Int J Circumpolar Health. 2019;78(2). Available from: <https://doi.org/10.1080/22423982.2018.1506213>

202. Morrison C, Fox K. Permanency through wabanaki eyes: A narrative perspective from “The People Who Live Where the Sun Rises.” Child Welfare. 2010;89(1):103-23.

203. Dombrowski K, Khan B, Moses J, Channell E, Dombrowski N. Network sampling of social divisions in a rural Inuit community. Identities. 2014;21(2):134–51. Available from: <https://doi.org/10.1080/1070289X.2013.854718>

204. Moses J, Khan B, Gauthier GR, Ponizovsky V, Dombrowski K. Confounding Culture: Drinking, Country Food Sharing, and Traditional Knowledge Networks in a Labrador Inuit Community. Hum Organ. 2017;76(2):171–83. Available from: <https://doi.org/10.17730/0018-7259.76.2.171>

205. Johnston G, Vukic A, Parker S. Cultural understanding in the provision of supportive and palliative care: perspectives in relation to an indigenous population. BMJ Support Palliat Care. 2013;3(1):61–8. Available from: <https://doi.org/10.1136/bmjspcare-2011-000122>

206. Ninomiya MEM, Pollock NJ. Reconciling community-based Indigenous research and academic practices: Knowing principles is not always enough. Soc Sci Med. 2017;172:28–36. Available from: <https://doi.org/10.1016/j.socscimed.2016.11.007>

207. Liebenberg L, Sylliboy A, Davis-Ward D, Vincent A. Meaningful engagement of Indigenous youth in PAR: The role of community partnerships. Int J Qual Methods. 2017;16(1):1–11. Available from: <https://doi.org/10.1177/1609406917704095>

208. Rosol R, Huet C, Wood M, Lennie C, Osborne G, Egeland GM. Prevalence of affirmative responses to questions of food insecurity: International Polar Year Inuit Health Survey, 2007–2008. Int J Circumpolar Health. 2011;70(5):488–97. Available from: <https://doi.org/10.3402/ijch.v70i5.17862>

209. Saini M, Roche S, Papadopoulos A, Markwick N, Shiwak I, Flowers C, et al. Promoting Inuit health through a participatory whiteboard video. Can J Public Heal. 2020;111(1):50–9. Available from: <https://doi.org/10.17269/s41997-019-00189-1>

210. Ungar M, Clark S E, Kwong W-M, Makhnach A, Cameron C.A. Studying resilience across cultures. J Ethn Cult Divers Soc Work, 2005*;*14(1-4), 1-19. Available from: [https://doi.org/10.1300/J051v14n03_01](https://doi.org/10.1080/22423982.2018.1506213)

211. Harper, S L. Weather, water, and infectious gastrointestinal illness in the context of climate change in Nunatsiavut, Canada [Masters thesis]. University of Guelph; 2010
