# Supplementary material for: Are we walking the talk of participatory Indigenous health research? A scoping review of the literature in Atlantic Canada
Source: PLoS One. 2021 Jul 27;16(7):e0255265. doi: 10.1371/journal.pone.0255265 (PMC8315539; doi:10.1371/journal.pone.0255265)
Supplement: Attachment — Submitted filename: Response to Reviewers .docx [file pone.0255265.s004.docx]

Below you will find each of the requested edits, with our responses to those edits listed in *blue italics*. For ease of review, we have organized the edits as follows: Journal Requirements, General Comments, Additional References Added, followed by all major headings in manuscript.

JOURNAL REQUIREMENTS:

1. Please ensure that your manuscript meets PLOS ONE's style requirements, including those for file naming. The PLOS ONE style templates can be found at

https://journals.plos.org/plosone/s/file?id=wjVg/PLOSOne_formatting_sample_main_body.pdf and

[https://journals.plos.org/plosone/s/file?id=ba62/PLOSOne_formatting_sample_title_authors_affiliations.pdf](about:blank)

*We have now revised all components of the manuscript to abide by PLOS ONE’s style requirements.*

2. Please include a table summarizing the characteristics of studies included.

*This table has been included in a prior publication, so we have referenced that publication on line 117 and again on line 245 to direct readers to further information about the characteristics of the studies included.*

3. We note that you have indicated that data from this study are available upon request. PLOS only allows data to be available upon request if there are legal or ethical restrictions on sharing data publicly. For information on unacceptable data access restrictions, please see <http://journals.plos.org/plosone/s/data-availability#loc-unacceptable-data-access-restrictions>.

a) If there are ethical or legal restrictions on sharing a de-identified data set, please explain them in detail (e.g., data contain potentially identifying or sensitive patient information) and who has imposed them (e.g., an ethics committee). Please also provide contact information for a data access committee, ethics committee, or other institutional body to which data requests may be sent.

b) If there are no restrictions, please upload the minimal anonymized data set necessary to replicate your study findings as either Supporting Information files or to a stable, public repository and provide us with the relevant URLs, DOIs, or accession numbers. Please see http://www.bmj.com/content/340/bmj.c181.long for guidelines on how to de-identify and prepare clinical data for publication. For a list of acceptable repositories, please see http://journals.plos.org/plosone/s/data-availability#loc-recommended-repositories.

We will update your Data Availability statement on your behalf to reflect the information you provide.

*We have included all of the articles we drew upon for our analysis in Supporting File 3 (S3). We believe that including this information satisfies the above requirements.*

4. PLOS requires an ORCID iD for the corresponding author in Editorial Manager on papers submitted after December 6th, 2016. Please ensure that you have an ORCID iD and that it is validated in Editorial Manager. To do this, go to ‘Update my Information’ (in the upper left-hand corner of the main menu), and click on the Fetch/Validate link next to the ORCID field. This will take you to the ORCID site and allow you to create a new iD or authenticate a pre-existing iD in Editorial Manager. Please see the following video for instructions on linking an ORCID iD to your Editorial Manager account: <https://www.youtube.com/watch?v=_xcclfuvtxQ>

*The corresponding author’s ORCID ID is :* [*https://orcid.org/0000-0002-6719-4677*](https://orcid.org/0000-0002-6719-4677) *. This has been updated in the submission.*

5. We note that you currently have two different versions of tables 1 and 2 included in your manuscript, so that the tables can be differentiated can you please update the table titles (numbering) and in-text citations so that the second set of tables are numbered individually (and not the same as the previous numbers for Tables 1 to 2 already used).

*We have updated the table numbering in both the table titles, and in the in-text references to each table.*

6. Please include captions for your Supporting Information files at the end of your manuscript, and update any in-text citations to match accordingly. Please see our Supporting Information guidelines for more information: [http://journals.plos.org/plosone/s/supporting-information](about:blank).

*We have included captions for our Supporting Information files at the end of the manuscript, and our in-text citation on line 734 matches accordingly.*

While revising your submission, please upload your figure files to the Preflight Analysis and Conversion Engine (PACE) digital diagnostic tool, <https://pacev2.apexcovantage.com/>. PACE helps ensure that figures meet PLOS requirements. To use PACE, you must first register as a user. Registration is free. Then, login and navigate to the UPLOAD tab, where you will find detailed instructions on how to use the tool. If you encounter any issues or have any questions when using PACE, please email PLOS at [figures@plos.org](mailto:figures@plos.org). Please note that Supporting Information files do not need this step.

*Figures 1 & 2 have been uploaded into PACE and formatted according to PLOS requirements.*

GENERAL COMMENTS

- More straightforward writing could be used throughout.

*The manuscript has been edited with brevity in mind, and all of the suggested reviewer edits have been incorporated.*

- Although this scoping review was focused on Atlantic Canada, it does not standout in this way. It could be from anywhere. Why is this important for Atlantic Canada? It is possible by including an aggregate descriptive table of the studies conducted in Atlantic Canada, that something distinct might be highlighted to include in the discussion and results. Nevertheless, consideration of the recommendations in this scoping review by researchers and community should expand beyond the region.

*We have modified a section of the Introduction beginning on Line 125 to indicate the specific value of this research to Atlantic Canada. We also note the value of place-based research for Indigenous research generally, which offers value beyond Atlantic Canada for this type of geographically-bounded scoping review.*

*With regard to an aggregate descriptive table of the studies conducted in Atlantic Canada, this table has been included in a prior publication and so we have referenced that publication on line 117 and again on line 245 to direct readers to further information about the characteristics of the studies included.*

ADDITIONAL REFERENCES REQUESTED

- Although reference 5 is an interesting article about the involvement of Elders in HIV CBR more suitable references for this statement are needed.

*This sentence was removed so no reference was added.*

- Line 64: ‘…complex inquiry of methods;’ include references.

*References 5 and 6 were added to support this.*

- Line 79: references needed.

*This sentence was modified from its original structure, but a reference (13) has been provided for the new sentence.*

- Line 96: ‘…Western-based research paradigm.’ References needed.

*This sentence is no longer present within the manuscript due to structural changes.*

- Line 115: ‘…their communities…’ references needed.

*This sentence is no longer present within the manuscript due to structural changes.*

- Line 149: ‘…meet the needs of their communities.’ References needed.

*This sentence is no longer present so no references were added.*

- Line 147-168: Insufficiently referenced.

*These sections have been extensively restructured due to structural changes within the introduction.*

ABSTRACT

- Line 30-32, Introduction: If you present findings for ‘b’ first then switch (a) and (b).

*In response to a later comment about an inconsistency between the abstract and introduction, we have edited the abstract so that this sentence has been entirely changed; therefore the issue that is noted here is no longer a concern.*

From Reviewer #1: 1) Abstract, Lines 28-29: The statement “However, there are inconsistencies in how it is applied in practice, as well as how it is reported.”: This statement implies that the inconsistencies of participatory research is a bad thing but I would disagree with this presumption as the application of participatory research should and would vary based on numerous factors such as prior relationships, history, nature of the research, etc. Further, this orientation to participatory research -- being flexible and in context -- is reflected in what the authors discuss on page 4, lines 87-91, which makes the message of this manuscript a bit confusing. Truthfully, how a research project is conducted with an Indigenous community, is between the community and the outside researchers. If the partnership works – albeit doesn’t check off all of the boxes for gold standard participatory research – then that is OK. Clearly, not all Indigenous communities have the interest or resources to take on all of the responsibilities outlined.

*We have re-worded the abstract to avoid framing the study as if there is a single, correct way to conduct participatory research that is easily measured. Instead, we are intending to emphasize the importance of how these relationships and engagement processes are reported within the literature to accurately reflect the nuances of how a research relationship unfolded. We recognize this complexity and completely agree with this comment. We hope that we have accurately addressed it not only within the abstract, but throughout the manuscript.*

2) Abstract lines 28-29: With respect to the second point of this statement (inconsistencies on how it is reported) may warrant the creation of a reporting template as you see for other types of studies (qualitative studies: COREQ; RCTs: CONSORT).

*This is wonderful feedback, and we have incorporated this suggestion into recommendations on line 645-648.*

*.*

3) Abstract, Line 38: Please note that the word “data” is plural so replace “was” with “were” in its use (abstract, line 38)

*Noted and addressed.*

4) Abstract, Line 45: Unclear what “transformations at the institutional level” is about. Vague language.

*This has been edited out of the abstract upon re-writing.*

5) Overall, if the intent of this scoping review is not what is perceived from the language used in the abstract (see comment #1), then the introduction should be revised accordingly (i.e., not to suggest that that manuscripts reporting on participatory health-related research with Indigenous communities should be uniform and consistent across the board).

*Absolutely. We agree with this statement and have strived to edit the manuscript to reflect our intent: which is to focus on how community engagement is reported in the literature, not whether or not a study has definitively engaged with a community. This comment is addressed more substantively in the limitations section of the manuscript.

INTRODUCTION*

- More straightforward writing needed throughout. Introduction should be more succinct. A lot of content that is better suited for the discussion. Very interesting ideas, but a lot of ideas with insufficient elaboration on some and too much on others. Described below suggested shifting some text to discussions or writing more succinctly.

*Thank you for this comment. We have revised the introduction of the manuscript substantially from the previous version. Some of the content has been moved to the discussion and the overall argument as presented in the introduction has shifted.*

Be mindful of generalizations that supports pan-Indigenous notions. ‘Indigenous worldviews are grounded in the concepts of …’ versus writing ‘Some Indigenous worldviews…’ given that there is only one reference.

*We agree with this comment and have edited the manuscript accordingly to avoid any pan-Indigenous assertions.*

- Inuit means people so ensure that sentences do not read ‘Inuit People(s)’ which might read as ‘People Peoples.’

*Thank you for this comment. We reviewed the manuscript and located one instance where we had said “…First Nations, Inuit and Métis Peoples”. We have removed ‘peoples’.*

- Is participatory research being discussed as part of CBR, CBPR or even PAR? If so then participatory research may involves some or all of its principles and may not involve a shift in power. Describe or define your understanding of participatory research and support this with the literature.

*Thank you for offering this point of clarification. We have now included clarification for our use of participatory research as an umbrella term for several different collaborative research approaches, starting on line 71 through to line 96. In this same paragraph we have discussed the general characteristics of participatory research as opposed to prescriptive principles, to better illustrate how we are conceptualizing participatory research as supported by the literature.*

*We have also bolstered our paragraph on participatory research to clarify that, while there are indeed a number of principles outlined in the literature for participatory research specifically, these are not always easy to apply in practice. These changes can be found beginning on line 77.*

- Line 86: ‘…community-driven methods…’ was the word ‘methods’ supposed to be the word ‘research’. This is the first mention of methods.

*This was an error and has been corrected to ‘research’.*
- Line 89: ‘…in situated ways...’ what is meant by situated ways and does the sentence hold the same meaning if this is removed.

*This has been removed.*
- Line 91: ‘…participatory methods…’ are you intending to talk about a part of the research or simply the methods. Clarify.

*This was also an error. It has been corrected to ‘research’.*

- Line 106: ‘…, and/or organization.’ References needed. What does the term participatory imply that findings and [others] would be vetted by an Indigenous group? Earlier you described that there is a range of ways community could be involved. If this is your perspective leave it to the discussion where your perspective might be supported by your findings.

*We have now removed the wording that was previously included on line 106, and lines 83-96 now represent the reworking of the participatory description to address this.*

- Line 113: What are the principles of participatory engagement?

*Rather than listing all of the principles of participatory engagement for fear of being too prescriptive in our description of participatory research, we have instead discussed some of its broad characteristics on lines 83-96.*

- Line 115-126: Insufficiently referenced. This sounds like you are drawing conclusions or making inferences from your experiences or observations. Most of the information in this paragraph would be better suited for a discussion with some references to support it. Find an alternative and more succinct way to connect to the next paragraph which also includes several sentences that need to be referenced (line 127-128, line 128-132) and made more succinct or would be better suited for a discussion.

*Much of this has now been removed, and elements incorporated into the conclusion*

- Line 128: ‘Therefore, the other point…’ this sentence seems disconnected from the first sentence. Is it about capacity mentioned in the prior paragraph? I can see how it works to connect to the ACADRE and IMNPs but should be made to be more fluid.

*This comment has been addressed through a more general edit of the entire paragraph, starting on line 108.*

- Line 167: Does Two-Eyed Seeing enhance the decolonizing goals of community-driven, participatory research? How? If this is a perspective leave it for the discussion or cite seminal literature by Elders Albert or Murdena Marshall with/without Dr. Bartlett

*Reference to Two-Eyed Seeing has now been removed.*

- Line 170: align objectives with the abstract or align abstract objectives with the ones in the introduction because they do not convey the same message. Use similar wording and ensure that you will clearly be examining community engagement.

*This issue has now been addressed by a thorough edit of the abstract, and it is now aligned with the introduction.*

METHODS

- More straightforward writing needed.

*Thank you for this feedback, and we have modified our methods section to make the writing more concise.*

- Line 178-182: Remove the definition of scoping review, not necessary. Stick to specifying the details of the review process described in lines 183-184.

*The definition of scoping review has been removed and this section now begins with the details of the review process, beginning on line 148.*

- Line 184: ‘It is important to acknowledge …’ include in the limitations section of a discussion.

*This has been moved to the limitations section on line 690.*

- Line 203: Is the review team the authors? If so say ‘We worked with …’

*We have edited throughout to indicate third person references when we are referring to ourselves.*

- Line 204: Did the broad keyword terms fall under the umbrella of the concepts of interest? If so, connect this.

*We have now made this connection between keywords and broad concepts of interest on line 162.*

- How was the ‘Native Friendship Centres’ included in the search strategy? What does this mean?

*Upon the guidance of our librarians, ‘Native Friendship Centres’ was included a search term (i.e. a keyword) because they have housed and authored Indigenous research in the region, and we wanted to ensure that their research was not omitted from our results. We have revised the sentence on lines 169-171 to clarify how it was included in our search strategy.*

- Line 221-223: Unnecessary to include the information in parenthesis for the database. For example, remove EBSCO, Elsevier, etc….

*The information in brackets has now been removed on lines 179-186.*

- Line 226-229: No need to include the search dates except for the last common search date (June 2020).

*This has been edited to reflect only the final search date (line 184).*

- Table 1 could be a supplementary table.

*We have removed Table 1 and have included it instead as a Supporting Information file. It is now referenced in-text as S1 Table on line 165.*

- Polish sections. For example, ‘… the following inclusion criteria if the study(ies): (1) included a focus…’ (line 241). There are multiple studies so no need to write ‘study(ies)’ just write studies. Also, this is a list of inclusion criteria so no need to write ‘included….’

*We have changed the wording to “…the following inclusion criteria if the studies: (1) focused on…” on line 240-1.*

- Line 245: Exclusion criteria are also not the opposite of inclusion criteria ‘…did not draw conclusions relating to Indigenous Peoples in Atlantic Canada.’ Could be removed because we already know you are going to exclude this based on your inclusion criteria.

*All exclusion criteria that was the inverse of our inclusion criteria has been removed. Our exclusion criteria has been updated on line2 194-97:*

- Line 247-249: Be more succinct.
- Line 250-252: Be more succinct. You have already written about title and abstract screening and following with full-text screening information, yet you have started this sentence by writing about title and abstract screening. Just start with full-text screening.

*Both of these edits have been made by doing a thorough edit of this section for succinctness and clarity.*

- Add a sub-heading for the ‘charting and analysis’ section.

*As noted below, we have added sub-headings according to PRISMA guidelines, which includes a ‘data charting’ section and a ‘synthesis of results’ section for our analysis.*

- Line 290: ‘We understand that the very…’ if you remove the word very does it hold the same meaning.

*The word “very” has been removed.*

- Line 302: Figure 1 is listed, but on page 23 of 68 is written figure 3. Have you inserted where to include all figures or only figure 3? Be consistent. Also, I do not see a figure 3 in your submission.

*Thank you for identifying this error. All of the figures have now been appropriately marked in the text, and the figure on line 240 has been changed to read ‘Figure 1’.*

RESULTS

- Line 302: Use PRISMA for scoping reviews: <http://prisma-statement.org/Extensions/ScopingReviews>

*Thank you for pointing us to this resource. We have indeed followed the PRISMA checklist for scoping reviews, however we have modified our headings on lines 159, 178, 188, 205, 215 to reflect those listed in that checklist, for clarity. Some reporting items were not included in the manuscript, including (1) the inclusion of a protocol (item 5) because we did not register or publish one; (2) the critical appraisal of included sources of evidence (item 16), as our objective was to look at the breadth of Indigenous health research in the Atlantic region as opposed to the quality of evidence reported in the literature; (3) characteristics of sources of evidence (item 15); and results of individual sources of evidence (item 17). The latter two (items 15 and 17) were not included due to the normative vs empirical nature of our scoping review (i.e., it scoped the terrain of Indigenous health research and how it was undertaken rather than empirical evidence from those studies). Finally, rather than including the search strategy for one database, we’ve included all keywords used in our search in S1 Table 1, which were modified for the specific needs of each database (details of which are outlined in our Methods section).*

*Figure 1 is the PRISMA flow diagram which outlines our selection of sources of evidence (PRISMA checklist item #14). We used the software Covidence to review the yielded articles at each stage of the screening process, and unfortunately, Covidence does not offer an option to indicate the reasoning for exclusion at the title/abstract screening stage (this option only exists at the full-text screening stage). Thus, in our PRIMSA flow diagram, we have included the reason for exclusion at the full-text screening stage (i.e. how many articles were excluded for what reason), but not at the title/abstract stage given the limitations of our chosen software. However, all screening was undertaken based on the inclusion/exclusion criteria we collaboratively developed a priori, which are listed in our Methods section.*

- Line 314: ‘92’ initially seems like a reference. Write to the first decimal place and use ‘n’ (n=92, 54.6%) and do so for all other numbers.

*We have now added ‘n’ before indicated the number of articles in the paragraph beginning on line 255, and have written the percentages to the first decimal place throughout the article, including the tables.*

- Line 319: ‘…reported engaging…’ vs ‘…reported engagement …’. Engagement sounds better.

*This has been edited throughout this paragraph to ‘engagement’ beginning on line 255 and throughout this paragraph.*

- Table 3: ‘All of the aspects’ vs ‘All aspects’ align with heading in 4th column. Verify numbers with the table with the numbers in the text. In the table n=92 is 50.53% whereas in the text n=92 is 54.55%. ‘Combination 2 or more’ should also be more aligned with all other column headings. For example, the table title includes the word engagement and so the column headings could be: All aspects, Two or more aspects, One aspect, None. ‘CBPR stated’ no need to say ‘stated’. ‘used’ in the last row no need to include this. Clean up the table.

*Column headings changed as recommended. Numbers have been reviewed and fixed. All column headings have been moved to center. The word “stated” after CBPR and “used” after Indigenous methodology/epistemology/ontology have been removed.*

- Figure 2: This figure is about engagement therefore leave this in the figure title and remove from x axis labels. For example, x axis labels: All aspects, Research design, Data collection, Data analysis, Development of research question, Dissemination of findings, None. Ensure that there is no ‘…’ in the labels because it is difficult to determine what some of the ‘…’ are because they are not all written in the text. There should be no ‘…’ in figure labels. ‘Stages of the Research Process’ also sounds better than ‘Research Stage’. In general, improve the esthetics of the figure and consider changing the color of the bars from blue to black because it will likely be printed in black and white.

*Thank you for this feedback. These changes have been addressed in Fig 2.*

- Consider changing ‘in a number of ways’ to ‘in several ways’ to diversify language a bit. Some wordsmithing and proofreading: ‘prior to the initiation of the research’ vs ‘prior to initiating the research’.

*Completed, on page 292.*

- Line 353: no need to write ‘etc’ since you have already written ‘e.g.’ for example so we know as a reader that the list is incomplete and in theory more could be added. Use ‘etc.’ sparingly.

*The use of ‘etc.’ has now been removed on line 297.*

- Line 378: Spell out OCAP upon first use since the audience for this journal may not be familiar with OCAP which is also trademarked so ensure this is indicated. CBPR should also be spelled out upon first use.

*Completed, on line 322-3.*

- Example of being more succinct with changes included in line 378: ‘Yet, with the breadth of articles that used OCAP principles in undertaking ethical Indigenous health research,35,39,64,70,74-76,82-91 only one study explicitly gave recognition to communities as ‘owners’ of the research enterprise.’ The following sentence includes ‘… grant proposal in collaboration with one another.’ ‘with one another’ could be deleted without losing the meaning of the sentence. Also, this sentence is very long so split it in half.

*Completed*

- Another example of writing in a more straightforward way, line 391: ‘Studies engaged communities in several aspects of the research process ….’ The following sentence could also be written in a more succinct way.

*Completed.*

- Some questions I had about the research was some basic descriptive information of the studies. Given there are a lot of studies just some basic aggregate information would have been interesting such as the number of studies per province (4 or 5 regions by separating NFLD and Labrador), specific Indigenous groups, quantitative or qualitative or both for example.

*This information has been included in a paper that is currently in-press in Social Sciences & Medicine:* [*https://www.sciencedirect.com/science/article/pii/S0277953621002793#fig2*](https://www.sciencedirect.com/science/article/pii/S0277953621002793#fig2)

*We have now referenced this article to point readers to descriptive information about the studies reviewed, by adding the following new text on lines 244-5.*

DISCUSSION
- Line 644: ‘The literature review…’ are you referring to your literature review. If so write ‘Our literature review…’

*Completed, on line 534.*
 
6) Concerned that this study is attempting to answer questions (whether or how) solely based on the literature while how Indigenous health research is conducted does not always find itself in the literature nor does the literature about such studies offer the degree of detail with respect to the participatory elements. That is, not reporting in a research article whether/how the Indigenous community was engaged in the study does not mean the Indigenous community was not engaged (e.g., lines 316-317). Thus, the tool of a scoping review appears to be inadequate to answer the whether or how research teams are actually enacting participatory and/or Indigenous-led processes. In fact, the irony is that the authors of this scoping review are criticizing the Ivory tower / Western ways while taking an Ivory tower / Western way approach itself. Thus, the overall message of this article is very confusing.

*Our limitations section alluded to this, however we have expanded our limitations to include those of Western-based literature reviews for capturing Indigenous knowledge on lines 690-708:*

*“Third, it is important to acknowledge the limitations of Western-based literature reviews in fully capturing Indigenous knowledge. According to Mi’kmaq scholar, Dr. Marie Battiste, conducting a literature review on Indigenous knowledge is “an oxymoron because Indigenous knowledge is typically embedded in the cumulative experiences and teachings of Indigenous peoples rather than the library”^27 (p. 2)^. We therefore recognize that in some ways, we are critiquing the very Western scientific approach we are assuming. However, we hold that, despite its imperfections, a scoping review provides a necessary snapshot of a broad body of work, so we can identify areas for further inquiry and improvement.”*

While the rigor detailed in the scoping reviews methodology is strong, the framing of this study needs to be revised in light of the concerns mentioned.

*Our team discussed this at length and have re-worded the framing of the study accordingly. We feel this offers a substantial contribution to the existing literature because if engagement with Indigenous communities is deemed an essential aspect of undertaking this type of research, but it is not required for being published within peer-reviewed literature, then there is an academic imperative to improve the reporting of Indigenous health research writ large.*

*Our wording in the limitations section (lines 679-685) is as follows:*

*“It is also important to acknowledge that our intent was not to assess definitively whether a study was or was not participatory, but rather, to assess whether and how the study reported this within the peer-reviewed literature. This is an important distinction, since it acknowledges that responsibility for reporting levels of community engagements rests not solely with the authors of studies, but also with the editorial decisions made by journals as to whether or not Indigenous engagement is a necessary reporting requirement.”*
